# Effect of Propagation Systems and Indole-3-Butyric Acid Potassium Salt (K-IBA) Concentrations on the Propagation of Peach Rootstocks by Stem Cuttings

**DOI:** 10.3390/plants10061151

**Published:** 2021-06-06

**Authors:** Ricardo A. Lesmes-Vesga, José X. Chaparro, Ali Sarkhosh, Mark A. Ritenour, Liliana M. Cano, Lorenzo Rossi

**Affiliations:** 1 Horticultural Sciences Department, Indian River Research and Education Center, Institute of Food and Agricultural Sciences, University of Florida, Fort Pierce, FL 34945, USA; ricardolesmes@ufl.edu (R.A.L.-V.); ritenour@ufl.edu (M.A.R.); 2 Horticultural Sciences Department, Institute of Food and Agricultural Sciences, University of Florida, Gainesville, FL 32603, USA; jaguey58@ufl.edu (J.X.C.); sarkhosha@ufl.edu (A.S.); 3 Plant Pathology Department, Indian River Research and Education Center, Institute of Food and Agricultural Sciences, University of Florida, Fort Pierce, FL 34945, USA; lmcano@ufl.edu

**Keywords:** vegetative propagation, *Prunus*, aeroponics, rooting, auxins

## Abstract

Traditionally, peach rootstocks are propagated by seeds due to their high availability, low cost, and easy storage and handling. However, stem cuttings allow the propagation of interspecific hybrids and keep the genetic uniformity of heterozygous genotypes. This study compared the effect of four different concentrations of K-IBA (indole-3-butyric acid potassium salt) on softwood cuttings of three peach backcrosses (peach × (peach × almond)) for rootstock propagation in two propagation systems: aeroponics and germination trays. The four concentrations of K-IBA applied were: 0.0% (*w/v*) as a control, 0.1% (*w/v*), 0.2% (*w/v*), and 0.4% (*w/v*). Data were collected on the survival rate (%), rooting rate (%), and root growth parameters. The relevance of auxin for peach cuttings rooting was evidenced. K-IBA at 0.2% showed the best rooting effect for peach softwood cuttings, evidenced by its high rooting rate and higher survival rate. K-IBA at 0.4% and 0.2% produced the highest number of adventitious roots. The highest root growth parameters were obtained in germination trays, confirming the suitability of this system for root growth. However, aeroponics was demonstrated to be as efficient as the traditional germination trays for the rooting of peach cuttings, allowing for a more controlled environment with a better use of resources.

## 1. Introduction

Peach scion cultivars (*Prunus persica* (L.) Batsch) are typically propagated on rootstocks for commercial production. Grafted plants offer numerous advantages over self-rooted plants, such as biotic [1,2] and abiotic plant stress resistance [3], enhanced field performance (yield, earliness), and improved horticultural management of orchards [4,5]. The rootstock can reduce canopy growth and size by influencing scion vigor, reducing production costs, and allowing higher planting densities [6]. Additionally, rootstocks affect positive precocity, crop efficiency, fruit size, and quality, among other traits [7,8]. Consequently, the choice of the appropriate rootstock is as important as scion selection.

Currently, seedling rootstocks predominate for peach propagation in the United States [4]. Seedlings have been used because of the availability of inexpensive seeds and the ease of sexual propagation compared to cuttings [9,10]. However, seed propagation may result in heterogeneity, with trees dissimilar from the mother tree and the consequent loss of desirable genetic traits due to pollen contamination (outcrossing events), which in the long term compromises the productivity of orchards [11,12].

The current trend in stone fruit production is for propagating rootstocks by rooting stem cuttings, excluding the chances of genetic variability and reducing time needing for seedling production [13,14]. However, despite the large number of studies of *Prunus* rootstocks, there is still limited information about clonal propagation using stem cuttings [15].

The rooting ability of cuttings is influenced by several factors, such as the genetic background of the rootstock, their horticultural management and nutritional status, the age of the stock plant, the cutting collection season, the endogenous content of photoassimilates and hormones, the type of cutting, the environmental manipulation of cuttings, the rooting media, and the hormonal treatment of the tissues [16,17,18,19,20,21]. In addition, the propagation of stone fruit using cuttings faces challenges from the diverse responses of *Prunus* spp. and cultivars to the plant growth regulator treatments. Generally, softwood cuttings of almond (*Prunus dulcis* (Mill.) D.A. Webb) are recalcitrant to root, whereas peach has a higher rooting rate and almond x peach hybrids show intermediate rooting [21,22,23].

Indole-3-butyric acid (IBA) is the most extensively used auxin for peach rootstock propagation by cuttings because of its role in enhancing root initiation and the number, quality, and uniformity of roots [19]. The peach varieties present successful rooting rates when treated with indole-3-butyric acid (IBA) at concentrations between 0.1% and 0.4% (*w/v*), with reported 0.2% (*w/v*) being as the optimal dosage [21,22,23].

Traditionally, peach rootstocks’ propagation by softwood cuttings is established in substrate media, with successful survival, rooting rate, and growth [14]. Although the overhead misting of cuttings inserted into a substrate is standard, leafy stem cuttings of woody plants can be propagated in environments that provide mist to the cuttings’ basal ends, with or without overhead mist [24]. This is the case in aeroponic systems, an interesting alternative for rooting peach stem cuttings [19]. Aeroponics consist of an air-water culture where plants are supplied with a nutrient solution by misting or fogging their bare roots. In addition, the use of fertilizers and water in these systems is more efficient due to their reuse since they are closed systems [25]. This technique provides a suitable balance between water and oxygen for rooting cuttings, enables the production of out-of-season trees, induces precocity, lowers disease and pest occurrence, and optimizes physical space [26,27]. Furthermore, aeroponics has been extensively used as a research tool for many difficult-to-propagate plant species [28], such as yam (*Dioscorea* spp.) [29,30], cut rose (*Rosa hybrida* L.) [31], and tamarisk (*Tamarix aphylla* (L.) H. Karst.) [32].

In traditional propagation systems, rooting rates are usually reported to be above 50% in peach semi-hard and hard cuttings treated with IBA in a concentration range of 0.1 to 0.4% (*w/v*) [33,34]. On the other hand, the rooting percentage of cuttings of species such as the myrobalan plum ‘Myrobalan 29C’ (*Prunus cerasifera* Ehrh.) and the peach × almond ‘GF 677′, treated with IBA at 0.2% (*w/v*), present contrasting rooting rates of 72% and 23%, respectively [35]. As of the time of writing, there have been few reports on the use of aeroponics to propagate *Prunus* spp. Coston et al. [24] demonstrated the successful rooting of semi-hardwood peach cuttings under aeroponic systems complemented with misting for the aerial portion of the cuttings. The objective of this study was to evaluate the rooting and performance of different peach softwood cuttings in two different propagation systems under the effect of four different concentrations of indole-3-butyric acid potassium salt (K-IBA).

## 2. Results

**Figure 1:**
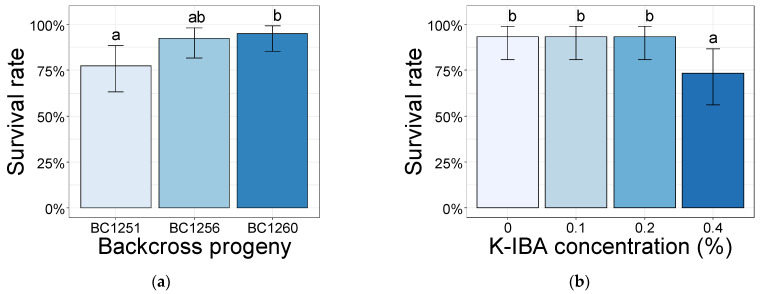
Survival rate (%) of the cuttings obtained from the peach backcross progenies BC1251, BC1256, and BC1260 (**a**), and the K-IBA concentration treatments applied at 0.0%, 0.1%, 0.2%, and 0.4% (*w/v*) (**b**). Data were calculated after 35 days of rooting. Bars bearing the different letters are significantly different at *p* ≤ 0.05.

After five weeks, the survival rate of the cuttings was above 73% for all of the softwood cuttings with non-significant differences between the aeroponic systems (91.7%) and the germination trays (85.0%) (Table S1). However, the survival rate of the backcross BC1251 progeny (77.5%) was significantly lower than that of backcross BC1260 (95%) (Figure 1a). The peach backcrosses in both propagation systems treated with K-IBA at 0.4% showed a significantly lower survival rate (73.3%) than the other K-IBA concentrations (0.0%, 0.1%, and 0.2%), which reported a rate of 93.3% (Figure 1b).

**Figure 2:**
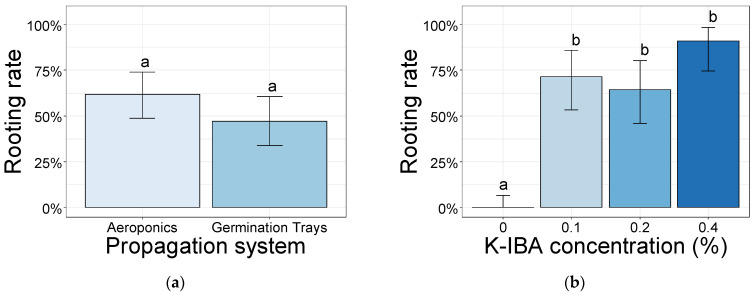

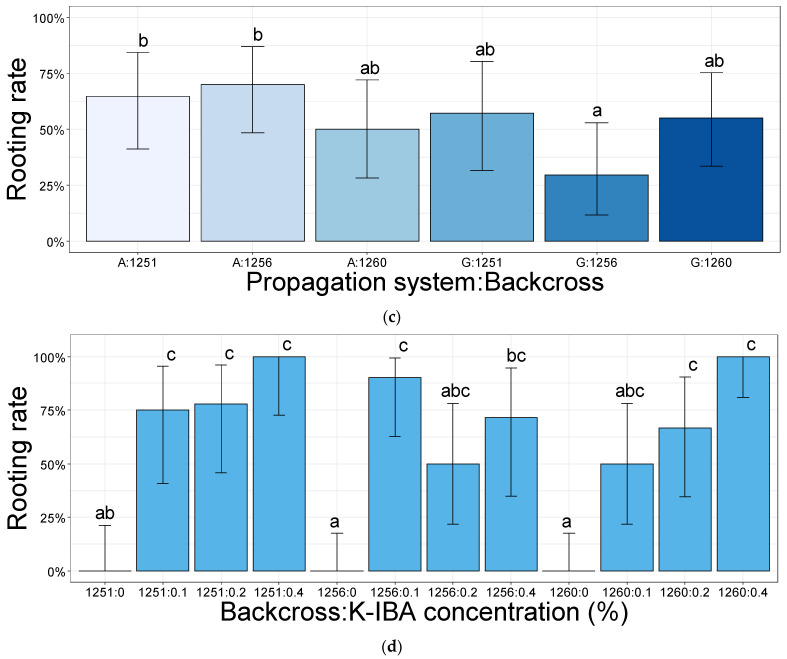
Rooting rate (%) of the softwood cuttings in both propagation systems (**a**) and treated with K-IBA at different concentrations (0.0%, 0.1%, 0.2%, and 0.4% (*w/v*)) (**b**). Rooting rate for the interaction propagation system: backcross progenies (1251 = BC1251, 1256 = BC1256, and 1260 = BC1260. A = Aeroponics systems, G = Germination trays). (**c**) Backcross progenies: K-IBA concentration (%) (**d**). Data were calculated after 35 days of rooting. Bars bearing the different letters are significantly different at *p* ≤ 0.05.

The rooting rate was higher under the aeroponic systems (61.8%) with a non-significant difference compared with the germination trays (47.1%) (Figure 2a). No rooting was observed in the cuttings treated with the control (K-IBA at 0.0%), whereas there were non-significant differences between the other K-IBA concentrations (Figure 2b), which showed rooting rates of above 64%. The backcross BC1256 presented a significantly higher rooting rate under aeroponic systems (70.0%) compared with the germination trays (29.4%) (Figure 2c). The aeroponic systems showed higher rooting rates for the BC1251 (64.7%) and BC1260 (50.0%) compared with the germination trays with non-significant differences, where BC1251 and BC1260 showed 57.1% and 55.0% rootings, respectively (Figure 2c). The backcross BC1251, as well as BC1260, showed a tendency to reach a higher rooting rate (%) as the K-IBA concentration increased from 0.1% to 0.4%, up to 100%. Conversely, BC1256 tended to reach a higher rooting rate (90%) when treated with K-IBA at 0.1% than with other K-IBA concentrations (Figure 2d).

**Figure 3:**
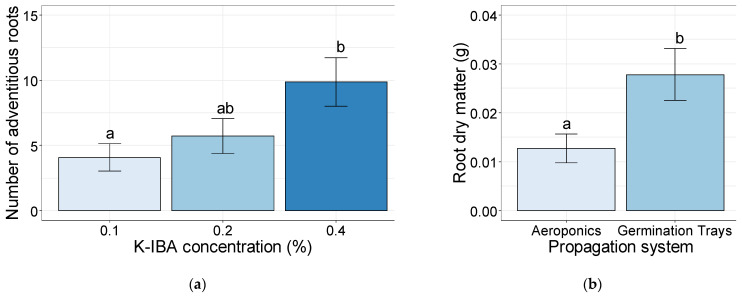
Number of adventitious roots of the softwood cuttings from the backcross progenies BC1251, BC1256, and BC1260, treated with K-IBA at 0.1%, 0.2%, and 0.4% (*w/v*) (**a**), and their dry matter (g) in both propagation systems (A = Aeroponics systems, G = Germination trays) (**b**). Data were calculated after 35 days of rooting. Bars bearing the different letters are significantly different at *p* ≤ 0.05.

There were non-significant differences in the number of adventitious roots between the propagation systems, backcross progenies, and their interactions (Table S2). However, the softwood cuttings treated with K-IBA at 0.4% showed a higher number of adventitious roots (>9) than those treated with K-IBA at 0.1% (>4), but were followed by K-IBA at 0.2% (>5) with a non-significant difference (Figure 3a). The root dry matter of the cuttings rooted in germination trays was significantly higher (0.0278 g) than those obtained in aeroponic systems (0.0127 g) (Figure 3b).

**Figure 4:**
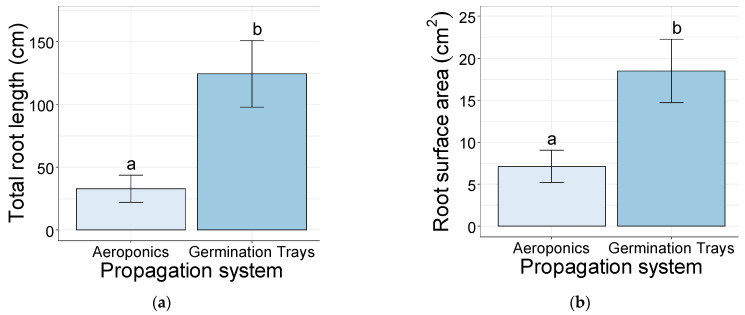
Total root length (cm) (**a**) and root surface area (cm^2^) (**b**) of the softwood cuttings after 35 days of rooting, established in aeroponics systems and germination trays. Bars bearing the different letters are significantly different at *p* ≤ 0.05.

The total root length of the softwood cuttings propagated in the germination trays was significantly higher (124.4 cm) than the ones propagated in the aeroponic systems (32.9 cm) (Figure 4a). Additionally, the softwood cuttings propagated in the germination trays reached a surface area significantly higher (18.47 cm^2^) than the ones propagated in the aeroponic systems (7.14 cm^2^) (Figure 4b).

**Figure 5:**
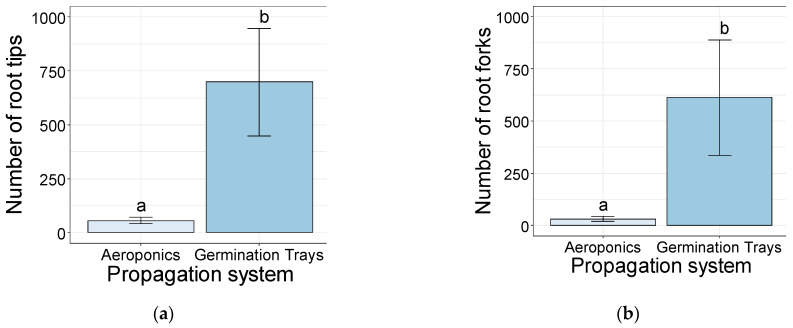
Number of roots tips (**a**) and number of root forks (**b**) of the softwood cuttings after 35 days of rooting, established in aeroponics systems and germination trays. Bars bearing the different letters are significantly different at *p* ≤ 0.05.

The softwood cuttings rooted in the germination trays showed a significantly higher number of root tips (698) than the ones in the aeroponic systems (56) (Figure 5a). Additionally, the number of root forks was significantly higher in the germination trays (>612) than in the aeroponic systems (>32) (Figure 5b).

**Figure 6:**
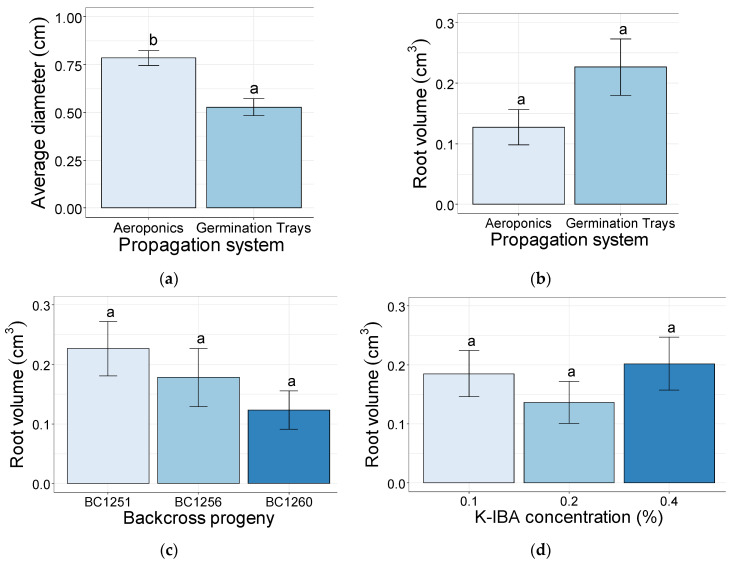
Root average diameter (cm) after 35 days of rooting of the softwood cuttings rooted in aeroponics systems and germination trays (**a**) and their root volume (cm^3^) comparing propagation systems (**b**), backcross progenies (**c**), and K-IBA concentrations (**d**). Bars bearing the different letters are significantly different at *p* ≤ 0.05.

The average root diameter of the softwood cuttings rooted in the aeroponic systems was significantly higher (0.78 cm) than the ones in the aeroponic systems (0.53 cm) (Figure 6a). Conversely, there were non-significant differences of root volume between softwood cuttings rooted in the aeroponic systems (0.13 cm^3^) and the ones in the germination trays (0.23 cm^3^) (Figure 6b), as well as when comparing the backcross progenies (Figure 6c) and the K-IBA applied at different concentrations (Figure 6d).

## 3. Discussion

The cuttings’ survival rate was high, with non-significant differences between both propagation systems, demonstrating them to be equally efficient in allowing the environment to keep healthy cuttings. This is relevant given that the propagation system, as well as the rooting media, are relevant for cutting survival and rooting success in *Prunus* [36,37,38]. The survival rate was also high for all of the backcross progenies, being consistent with survival rates reported for the ‘Flordaguard’ peach [39]. However, significant differences in the cutting survival rate between the backcross progenies BC1251 and BC1260 demonstrate that not all of the plant materials perform the same. It is worthy to mention that the BC1260 progeny from this study has a higher percentage of ‘Flordaguard’ pedigree due its female parental line. The lower survival rate observed for the cuttings treated with K-IBA at 0.4% demonstrates that growth regulators such as auxins have to be applied in the appropriate concentrations. These results are consistent with the findings of Kaur [40] in ‘Flordaguard’ rootstock, where the survival rate decreases dramatically from the concentration 0.4% of IBA (40.25% decrease). The application of IBA at higher concentrations beyond the optimum reduced cutting survival rates as reported by other authors [41,42]. This inhibitory effect due to high IBA concentrations may be explained by the toxicity of the Potassium (K^+^) ions from the auxin solution, which can damage epidermal tissues and adjacent cells [43]. However, it is worth mentioning that the rooting ability of IBA, as well as the optimum concentration, depend on the plant species [44]. Within the *Prunus* genus, these can vary depending upon the species and even the variety [21,45].

The aeroponic systems demonstrated to be as or more effective than the germination trays to root *Prunus* species, such as the plant materials used for this experiment. Advantages and weaknesses were observed in both propagation systems. The substrate moisture provides a hydration backup in case of a lack of watering for longer periods, whereas in aeroponics, there is a lack of a buffer around the roots for water and nutrients. However, aeroponics required easier handling and less labor than the germination trays. The germination trays required more sources and labor, such as substrate mixing, autoclaving, filling trays, and fungicide drenching. The aeroponics provided a cleaner environment for cuttings rooting, with no substrate and less labor. The closed spraying system of the aeroponics allows the reuse of water and nutrients. The aeroponics did not require a misting system or a plastic dome to keep the cuttings hydrated. Conversely, the germination trays required a plastic dome covering and daily spraying throughout all of the experiment to keep cuttings hydrated. On the other hand, the aeroponics allow access to clean intact roots for direct observation of the cuttings rooting progress and visualize the status of the bare roots in vivo [46].

The lack of rooting in the cuttings treated with K-IBA at 0.0% demonstrates the essential role of IBA for rooting cuttings and vegetative propagation in *Prunus*. The contrasting values of survival rate and rooting rate between treatments with K-IBA at 0.0% and 0.4% confirmed the relevance of auxin treatments for cuttings, despite an efficient propagation system that guarantees their survival. These results confirmed the findings of other research about the requirement for rooting hormones for the successful propagation by cuttings in cultivated species [19,47,48,49]. In addition, the rooting rates in the 0.1%, 0.2%, and 0.4% K-IBA treatments are similar to the reports of Tsipouridis et al. [50], who found that IBA applied at 0.2% (*w/v*) conferred excellent rooting ability to peach semi-hardwood cuttings, within a range of peach rootstocks varieties, such as ‘Guardian^®^’, which has been reported to have an optimum IBA concentration of 0.3% [15].

The applied K-IBA concentration was the primary factor influencing the number of adventitious roots, with there being no significant differences between backcross progenies or propagation systems. The number of adventitious roots was not significantly different between the cuttings treated with K-IBA at 0.2% and 0.4%. These results are in accordance with other authors’ findings in the *Prunus* species [21,41,42].

The root growth-related parameters confirmed the propagation system as an integral part of the propagation method by directly influencing the root traits measured in this study [51]. Parameters such as dry matter (g), total length (cm), and surface area (cm^2^) were significantly higher in the cuttings established in germination trays, confirming the superiority of potting mix for the root growth in cuttings. These results were consistent with the number of root tips and the number of root forks, which were influenced by the rooting system. Importantly, the germination trays allowed for significantly higher values compared with the aeroponic systems. Nevertheless, since the cuttings were not fertilized during the experiment, the higher values of these parameters in the germination trays could have been influenced by the breakdown of organic substrate, providing nutrients and root-promoting substances to the cuttings that are absent or in lower concentrations in the irrigation water of the aeroponic systems [52].

The higher root average diameter values (cm) from cuttings in aeroponic systems can be explained by the way WinRHIZO software estimates this parameter (see materials and methods). This is a characteristic root thickening response to aeroponics that has been demonstrated previously and is more pronounced than other soilless techniques such as hydroponics [53].

## 4. Materials and Methods

Plant material. Three different peach (*Prunus persica* (L.) Batsch) × (peach × almond (*Prunus dulcis* [Mill.] D.A. Webb)) backcross progenies—BC1251, BC1256, and BC1260—were used in this study (Table 1). The peach × almond parental selections (1251, 1256, and 1260) used to generate the backcross hybrids are highly resistant to the peach fungal gummosis caused by *Botryosphaeria dothidea*. The female parental line is ‘Flordaguard’, a peach rootstock variety highly resistant to root-knot nematodes (*Meloidogyne* spp.). The ‘Flordaguard’ peach was released by University of Florida breeders (Gainesville, FL, USA) in 1991 and is being used in other countries’ subtropical regions due its root-knot nematode resistance and other advantages, such as compatibility with all peach varieties, easy vegetative propagation, and readiness to graft in a shorter time than seedlings [40,54]. These materials segregate rootstock populations for peach production in Florida soils (USA) with contrasting edaphic features: Sandy Entisols of the Central Ridge and Wet Spodosols of the Flatwoods [55]. Leafy softwood cuttings were obtained from 1-year old stock plants of these backcrosses, grown under a plastic-covered greenhouse located in Fort Pierce, FL, USA at 27°25′34.2″ N–80°24′34.0″ W.

Rooting treatments. The cuttings had three leaves each and were cut 2 cm from the petiole. The surface of the leafy softwood cuttings were disinfected by immersion in sodium hypochlorite (0.75% *v/v*) for ten minutes, followed by a twenty-minute rinsing in distilled water. Softwood cuttings 10 to 12 cm long were cut under water to avoid embolism in vascular tissues. The basal portion of the cuttings were washed with spraying water for 24 h before the rooting treatment to prevent prunasin synthesis in the tissues [22] and to enhance their rooting ability [19,56]. Prior to rooting hormone treatments, a 2 cm-long incision was made at each cuttings’ base to enhance the rooting process [57,58]. The rooting hormone treatments consisted of immersing the base of the cuttings for 15 s in 4 different concentrations of indole-3-butyric acid potassium salt (K-IBA) dissolved in distilled water (*w/v*) previously autoclaved (121 °C for 90 min): 0.0% (*w/v*) as control, 0.1% (*w/v*), 0.2% (*w/v*), and 0.4% (*w/v*). K-IBA was used since its formulation enables the dilution of IBA in water with similar rooting induction without the negative effects of solutions made using alcohol [59], which increases the risk of tissue dehydration and burning, caused by the disintegration of cortical tissues, predisposing the cuttings’ base to a pathogen attack [19,60]. The rooting solutions were stored in the dark at 4 °C to avoid any chemical denaturation. A fertilizer was not supplied to any propagation system during the experiment.

**Figure 7:**
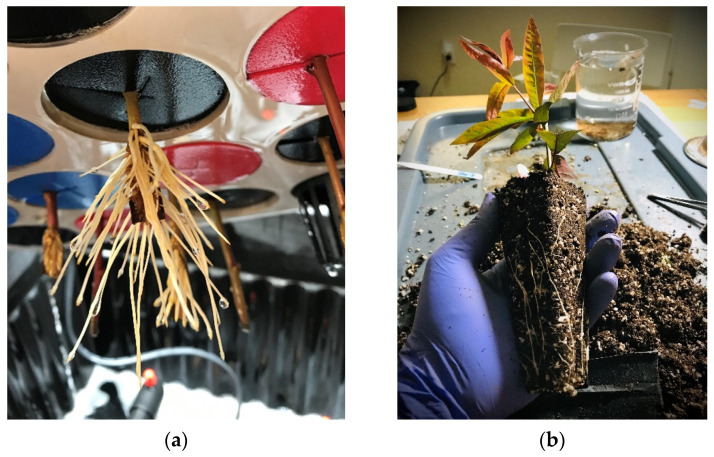
Samples of softwood cuttings from the peach backcross progenies rooted in the aeroponic systems (**a**) and in the germination trays (**b**).

Plant growing conditions. The leafy softwood cuttings were established in two propagation systems: germination trays and aeroponic systems (Figure 7). The germination trays (720700C SureRoots ^®^; T.O. Plastics, Inc.; Clearwater, MN, USA) consisted of star-shaped deep cell plugs (12.7 cm depth × 5.1 cm top width) containing a mixture (1:1) of potting mix sphagnum (Jolly Gardener^®^ Pro-Line C/20 Growing Mix; Jolly Gardener Products, Inc.; Poland Spring, ME, USA) and coarse perlite (Specialty Vermiculite Corp.; Pompano Beach, FL). The substrate mixture was autoclaved at 121 °C for 90 min. The cuttings in the germination trays were watered by manual spraying and covered with transparent plastic domes (Mondi TM; Vancouver, BC, Canada) of 17.8 cm height to maintain humidity. The aeroponic systems (Clone King Aeroponic Systems; Albuquerque, NM, USA) consisted in 10-L containers with 25 sites, a submersible pump (140 L/hour), and a spray manifold with misters (280-degree spray head, 0.4 mm diameter). The submersible pumps of the aeroponic systems worked continuously to provide water to the cuttings. The experiment was set under laboratory conditions with 23 °C and 65% relative humidity (RH), where a complementary light source was adapted to keep the photoperiod at 16 h–8 h (day-night).

**Figure 8:**
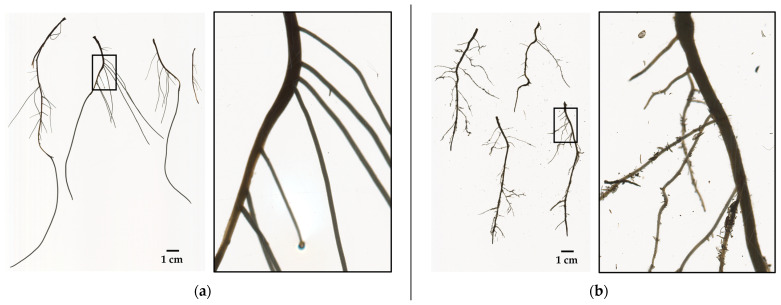
Samples of scanner-based images at 600 dpi (dots per inch). The bare root samples were of softwood cuttings from the backcross BC1256, treated with K-IBA at 0.1% (*w/v*), and rooted in one of the aeroponic systems (**a**) and in one of the germination trays (**b**).

Root measurements. Destructive sampling was carried out after five weeks for data collection of the survival rate (%), rooting rate (%), number of adventitious roots, and roots dry matter (g). Survival rate was based on the percentage of live cuttings per treatment at the moment of data collection [61]. Root growth parameters: total root length (cm), root surface area (cm^2^), average root diameter (cm), root volume (cm^3^), number of root tips, and number of root forks were measured on fresh roots by the analysis of scanner-based images. The roots were scanned by placing them in a Plexiglas tray (200 mm × 300 mm × 15 mm) with water in order to untangle the roots and minimize root overlap. The images were captured in TIFF (Tagged Image Format File) at a resolution of 600 dpi (dots per inch) (Figure 8). The image analysis was done using the root image analysis software, WinRHIZO Pro 2019 (Regent Instruments, Inc., Quebec City, Quebec, Canada). Noticeably, WinRHIZO software estimates the average diameter from the total projected root area (cm^2^) divided by the total length (cm) [62]. Afterwards, the dry matter (g) was estimated by placing the root samples in paper bags at 70 °C for 7 days.

**Figure 9:**
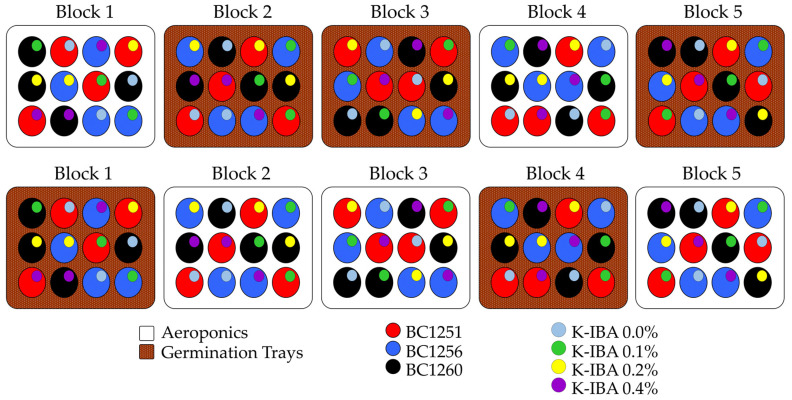
Scheme of the experiment setting under a split-plot experimental design with five replications, under laboratory conditions.

Experimental design and statistical analysis. The experiment was arranged in a split-plot design with five replications (Figure 9), using one cutting per replicate, where the propagation systems (germination trays vs. aeroponics) were the main plot factors, the K-IBA concentrations applied, and the backcross softwood cuttings were the subplot factors. The collected data were analyzed using a linear mixed model in the RStudio software version 4.0.4 (R, 2019), with a significance level of 0.05, and the means separation was done using the Tukey HSD (Honestly Significance Difference) test. Results are presented as the simple binomial proportions and the binomial likelihood ratio of 95% confidence intervals.

## 5. Conclusions

Exploring alternative systems for peach rootstock propagation are important due the implications that interesting findings may have for research and production applications. Moreover, identifying the appropriate rooting hormone treatment and the performance of aeroponics at such concentrations is useful for the study of promising plant materials concerning their clonal propagation. The application of IBA is essential for the successful rooting of peach × (peach × almond) backcross cuttings. The best auxin concentration treatment for cuttings rooting in both propagation systems was the K-IBA at 0.2%. Although the germination trays demonstrated higher results in most of the root growth parameters, aeroponic systems represent a promising alternative for the vegetative propagation of stone fruit rootstocks using softwood cuttings. The satisfactory performance and success of aeroponic systems improves propagation efficiency and facilitates root biology research. The aeroponic system allows the observation of callus formation, root induction, and bare root growth in real time, allowing the determination of the optimal moment for transplanting. Moreover, the efficiency of propagation research is enhanced by the easy manipulation of the rooted cuttings for scanning, measurement, and potting. In conclusion, the rooting of softwood peach cuttings when treated with K-IBA at 0.2% and established in aeroponic systems represents a valuable alternative to the traditional vegetative propagation method.

## Figures and Tables

**Table 1:** Interspecific peach × (peach × almond) backcross populations from which the leafy softwood cuttings were obtained.

Backcross	Female (Peach)	Male (Peach × Almond)
BC1251	‘R95654.16’	‘Fla. 97-47c’ × ‘Tardy-Nonpareil’ #1251
BC1256	‘R95654.16’	‘Fla. 97-42c’ × ‘Tardy-Nonpareil’ #1256
BC1260	‘R95654.16’	‘Flordaguard’ × ‘Tardy-Nonpareil’ #1260

## Data Availability

The data presented in this study are available on request from the corresponding author.

## Supplementary Materials

The following are available online at https://www.mdpi.com/article/10.3390/plants10061151/s1: Table S1: Statistical results of the survival rate (%) and rooting rate (%) from the peach backcrosses (BC1251, BC1256, and BC1260) for the factors (propagation systems, backcross progenies, K-IBA concentrations, and their interactions) that presented differences, grouped by letters. Table S2: Statistical results of the root growth parameters (number of adventitious roots, dry matter (g), length (cm), surface area (cm^2^), diameter (cm), volume (cm^3^), number of root tips, and number of root forks) from the peach backcrosses (BC1251, BC1256, and BC1260) for the factors (K-IBA concentrations, backcross progenies, and propagation systems) that presented differences, grouped by letters.
