# Supplementary material for: Effect of Propagation Systems and Indole-3-Butyric Acid Potassium Salt (K-IBA) Concentrations on the Propagation of Peach Rootstocks by Stem Cuttings
Source: Plants (Basel). 2021 Jun 6;10(6):1151. doi: 10.3390/plants10061151 (PMC8229110; doi:10.3390/plants10061151)
Supplement: Supplementary file 1 [file plants-10-01151-s001.zip › plants-1147296-supplementary.pdf]

**Table S1.** Statistical results of the survival rate (%) and rooting rate (%) from the peach backcrosses (BC1251, BC1256 and BC1260) for the factors (Propagation systems, backcrosses progenies, K-IBA concentrations, and their interactions) that presented differences, grouped by letters.

| Survival Rate (%) of backcrosses progenies | | | | |
|---|---|---|---|---|
| **Backcross progeny** | **Response** | **Lower** | **Upper** | **Group** |
| BC1251 | 77.5 | 63.10 | 88.49 | a |
| BC1256 | 92.5 | 81.69 | 98.08 | ab |
| BC1260 | 95.0 | 85.35 | 99.15 | b |
| **Survival Rate (%) of K-IBA concentrations** | | | | |
| **K-IBA concentration (%)** | **Response** | **Lower** | **Upper** | **Group** |
| 0.0 | 93.3 | 80.80 | 98.86 | b |
| 0.1 | 93.3 | 80.80 | 98.86 | b |
| 0.2 | 93.3 | 80.80 | 98.86 | b |
| 0.4 | 73.3 | 56.03 | 86.81 | a |
| **Survival Rate (%) of propagation systems (Not shown in the paper)** | | | | |
| **Propagation system** | **Response** | **Lower** | **Upper** | **Group** |
| Aeroponic systems | 91.7 | 82.93 | 96.93 | a |
| Germination trays | 85.0 | 74.58 | 92.49 | a |
| **Rooting (%) of propagation systems** | | | | |
| **Propagation system** | **Response** | **Lower** | **Upper** | **Group** |
| Aeroponic systems | 61.8 | 48.65 | 73.90 | a |
| Germination trays | 47.1 | 33.76 | 60.65 | a |
| **Rooting (%) of K-IBA concentrations** | | | | |
| **K-IBA concentration (%)** | **Response** | **Lower** | **Upper** | **Group** |
| 0.0 | 0.0 | 0.00 | 6.63 | a |
| 0.1 | 71.4 | 53.32 | 85.78 | b |
| 0.2 | 64.3 | 45.86 | 80.21 | b |
| 0.4 | 90.9 | 74.45 | 98.43 | b |
| **Rooting (%) of the interaction propagation system : backcross progeny** | | | | |
| **System : Backcross** | **Response** | **Lower** | **Upper** | **Group** |
| A : BC1251 | 64.7 | 41.09 | 84.18 | b |
| A : BC1256 | 70.0 | 48.35 | 86.82 | b |
| A : BC1260 | 50.0 | 28.08 | 71.92 | ab |
| G : BC1251 | 57.1 | 31.68 | 80.20 | ab |
| G : BC1256 | 29.4 | 11.70 | 52.90 | a |
| G : BC1260 | 55.0 | 33.59 | 75.20 | ab |
| **Rooting (%) of interaction backcross progeny : K-IBA concentration** | | | | |

| **Backcross : K-IBA (%)** | **Response** | **Lower** | **Upper** | **Group** |
|---|---|---|---|---|
| BC1251:0.0 | 0.0 | 0.00 | 21.34 | ab |
| BC1251:0.1 | 75.0 | 40.87 | 95.34 | c |
| BC1251:0.2 | 77.8 | 45.83 | 95.92 | c |
| BC1251:0.4 | 100.0 | 72.60 | 100.00 | c |
| BC1256:0.0 | 0.0 | 0.00 | 17.48 | a |
| BC1256:0.1 | 90.0 | 62.84 | 99.40 | c |
| BC1256:0.2 | 50.0 | 21.76 | 78.24 | abc |
| BC1256:0.4 | 71.4 | 35.02 | 94.58 | bc |
| BC1260:0.0 | 0.0 | 0.00 | 17.48 | a |
| BC1260:0.1 | 50.0 | 21.76 | 78.24 | abc |
| BC1260:0.2 | 66.7 | 34.54 | 90.45 | c |
| BC1260:0.4 | 100.0 | 80.78 | 100.00 | c |

**Rooting (%) of backcrosses progenies (Not shown in the paper)**

| **Backcross progeny** | **Response** | **Lower** | **Upper** | **Group** |
|---|---|---|---|---|
| BC1251 | 61.3 | 43.75 | 77.03 | a |
| BC1256 | 51.4 | 35.56 | 66.96 | a |
| BC1260 | 52.6 | 36.98 | 67.94 | a |

**Table S2.** Statistical results of the root growth parameters (Number of adventitious roots, dry matter (g), length (cm), surface area ($cm^2$), diameter (cm), volume ($cm^3$), number of root tips and number of root forks) from the peach backcrosses (BC1251, BC1256 and BC1260) for the factors (K-IBA concentrations, backcrosses progenies, and propagation systems) that presented differences, grouped by letters.

| **Number of adventitious roots of K-IBA concentrations** | | | | |
|---|---|---|---|---|
| **K-IBA concentration (%)** | **Response** | **Lower CL** | **Upper CL** | **Group** |
| 0.1 | 4.07 | 2.19 | 6.52 | a |
| 0.2 | 5.72 | 3.3 | 8.88 | ab |
| 0.4 | 9.85 | 6.42 | 14.02 | b |
| **Number of adventitious roots of propagation systems (Not shown in the paper)** | | | | |
| **Propagation system** | **Response** | **Lower CL** | **Upper CL** | **Group** |
| Aeroponic systems | 6.21 | 4.01 | 8.88 | a |
| Germination trays | 6.46 | 3.89 | 9.66 | a |
| **Number of adventitious roots of backcrosses progenies (Not shown in the paper)** | | | | |
| **Backcross progeny** | **Response** | **Lower CL** | **Upper CL** | **Group** |
| BC1251 | 9.17 | 6.07 | 12.90 | a |
| BC1256 | 5.52 | 2.63 | 9.48 | a |
| BC1260 | 4.71 | 2.65 | 7.34 | a |
| **Roots dry matter (g) of propagation systems** | | | | |
| **Propagation system** | **Response** | **Lower CL** | **Upper CL** | **Group** |
| Aeroponic systems | 0.0127 | 0.00659 | 0.0208 | a |
| Germination trays | 0.0278 | 0.0175 | 0.0405 | b |
| **Total roots length (cm) of propagation systems** | | | | |
| **Propagation system** | **Response** | **Lower CL** | **Upper CL** | **Group** |
| Aeroponic systems | 32.9 | 11.9 | 64.3 | a |
| Germination trays | 124.4 | 74 | 187.7 | b |
| **Roots surface area ($cm^2$) of propagation systems** | | | | |
| **Propagation system** | **Response** | **Lower CL** | **Upper CL** | **Group** |
| Aeroponic systems | 7.14 | 3.24 | 12.6 | a |
| Germination trays | 18.47 | 11.2 | 27.6 | b |
| **Number of root tips of propagation systems** | | | | |
| **Propagation system** | **Response** | **Lower CL** | **Upper CL** | **Group** |
| Aeroponic systems | 56 | 28.7 | 109 | a |
| Germination trays | 698 | 327.3 | 1488 | b |
| **Number of root forks of propagation systems** | | | | |
| **Propagation system** | **Response** | **Lower CL** | **Upper CL** | **Group** |
| Aeroponic systems | 32.1 | 12.8 | 78.5 | a |
| Germination trays | 612.1 | 231.8 | 1614.1 | b |

| Average root diameter (cm) of propagation systems | | | | |
|---|---|---|---|---|
| **Propagation system** | **Response** | **Lower CL** | **Upper CL** | **Group** |
| Aeroponic systems | 0.78 | 0.688 | 0.887 | b |
| Germination trays | 0.53 | 0.437 | 0.626 | a |

| Average root volume (cm³) of propagation systems | | | | |
|---|---|---|---|---|
| **Propagation system** | **Response** | **Lower CL** | **Upper CL** | **Group** |
| Aeroponic systems | 0.13 | 0.066 | 0.209 | a |
| Germination trays | 0.23 | 0.136 | 0.340 | a |

| Average root volume (cm³) of backcrosses progenies | | | | |
|---|---|---|---|---|
| **Backcross progeny** | **Response** | **Lower CL** | **Upper CL** | **Group** |
| BC1251 | 0.23 | 0.137 | 0.338 | a |
| BC1256 | 0.18 | 0.091 | 0.294 | a |
| BC1260 | 0.12 | 0.062 | 0.205 | a |

| Average root volume (cm³) of K-IBA concentrations | | | | |
|---|---|---|---|---|
| **K-IBA concentration (%)** | **Response** | **Lower CL** | **Upper CL** | **Group** |
| 0.1 | 0.19 | 0.108 | 0.282 | a |
| 0.2 | 0.14 | 0.070 | 0.224 | a |
| 0.4 | 0.20 | 0.117 | 0.309 | a |
